# Neck Pain: Do We Know Enough About the Sensorimotor Control System?

**DOI:** 10.3389/fncom.2022.946514

**Published:** 2022-07-15

**Authors:** Ning Qu, HaoChun Tian, Enrico De Martino, Bin Zhang

**Affiliations:** ^1^Department of Orthopedic Surgery, The First Affiliated Hospital of Nanchang University, Nanchang, China; ^2^Center for Neuroplasticity and Pain (CNAP), Department of Health Science and Technology, Faculty of Medicine, Aalborg University, Aalborg, Denmark; ^3^Aerospace Medicine and Rehabilitation Laboratory, Department of Sport, Exercise and Rehabilitation, Faculty of Health and Life Sciences, Northumbria University, Newcastle upon Tyne, United Kingdom

**Keywords:** neck pain, proprioception, sensorimotor control, neural plasticity, intervention

## Abstract

Neck pain is a worldwide health problem. Clarifying the etiology and providing effective interventions are challenging for the multifactorial nature of neck pain. As an essential component of cervical spine function, the sensorimotor control system has been extensively studied in both healthy and pathological conditions. Proprioceptive signals generated from cervical structures are crucial to normal cervical functions, and abnormal proprioception caused by neck pain leads to alterations in neural plasticity, cervical muscle recruitment and cervical kinematics. The long-term sensorimotor disturbance and maladaptive neural plasticity are supposed to contribute to the recurrence and chronicity of neck pain. Therefore, multiple clinical evaluations and treatments aiming at restoring the sensorimotor control system and neural plasticity have been proposed. This paper provides a short review on neck pain from perspectives of proprioception, sensorimotor control system, neural plasticity and potential interventions. Future research may need to clarify the molecular mechanism underlying proprioception and pain. The existing assessment methods of cervical proprioceptive impairment and corresponding treatments may need to be systematically reevaluated and standardized. Additionally, new precise motor parameters reflecting sensorimotor deficit and more effective interventions targeting the sensorimotor control system or neural plasticity are encouraged to be proposed.

## Introduction

Neck pain is one of the most commonly reported musculoskeletal disorders, causing a substantial economic burden to healthcare systems, absence from work, and compensations (Kazeminasab et al., 2022). Around 50% of the adult population experience at least one episode of neck pain during their lifetime, and neck pain ranks fourth in the leading causes of global disabilities (Fejer et al., 2006; Hoy et al., 2014). The main challenge in the long-term management of neck pain is to provide accurate diagnosis and effective therapies (Cohen, 2015; Vardeh et al., 2016). Neck pain is a multifactorial disease influenced by many biological, psychological, behavioral and social factors, making it challenging to identify the main contributors and their relevance to the consequences of neck pain (Kazeminasab et al., 2022). A large portion of neck pain patients is classified as non-specific since a clear pathoanatomical etiology of the neck pain is not detected (McLean et al., 2010; Misailidou et al., 2010), which makes therapies tend to focus on addressing the symptoms and the physical impairments of neck pain.

**FIGURE 1:**
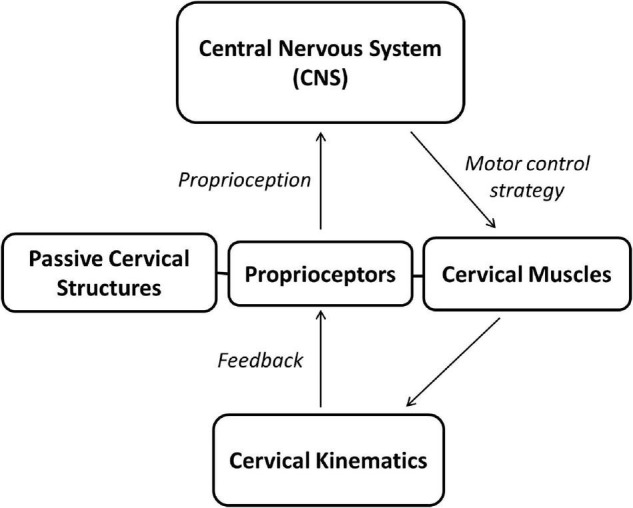
The cervical sensorimotor control system. The central neural system instantly processes proprioception generated from proprioceptors in cervical structures (i.e., cervical muscles and passive structures) and sends motor commends to cervical muscles to complete neck movements. The neck movements, in turn, could affect the proprioception generation.

Sensorimotor control system is a very important component of the cervical spine (Figure 1). The impaired proprioception and disturbance of the sensorimotor control system in neck pain have been extensively studied in previous research studies (Reddy et al., 2019; Asiri et al., 2021; Peng et al., 2021), and the long-term sensorimotor alteration and neural plasticity changes due to persistent proprioceptive deficit has been suggested to contribute to the recurrence and chronicity of neck pain (Hodges and Tucker, 2011; Röijezon et al., 2015; Kristjansson et al., 2016; Brumagne et al., 2019). This review presents a short update on proprioception of the cervical spine and impaired proprioception in patients with neck pain. First, the sensorimotor control system of the cervical spine is introduced to evaluate mechanisms underlying normal and neck pain conditions. Then, maladaptive neural plasticity will be discussed in chronic neck pain conditions, and, finally, interventions to manipulate the sensorimotor control system and maladaptive neural plasticity will be proposed.

## Proprioceptor and Proprioception

### Cervical Proprioception

Cervical proprioception refers to sensory information generated by muscle spindle, Golgi tendon organs (GTOs), joint receptors and cutaneous receptors, which located in muscle, tendon, joint capsules and skin, respectively (Hogervorst and Brand, 1998; Delhaye et al., 2018; Kröger, 2018). The constant sensory information, together with the vestibular and visual systems, ensures coordinated motor functions and rapid reaction of the neck to the surrounding environment (Proske and Gandevia, 2012; Kiehn, 2016). The proprioception plays a crucial role in maintaining posture and stability of the cervical joints during static and dynamic situations (Strimpakos, 2011; Proske and Gandevia, 2018). Extensive literature indicates that GTOs and muscle spindles mainly contribute to neck proprioception, while the contribution of joint and cutaneous receptors are minimal (Armstrong et al., 2008; van der Wal, 2009; Proske and Gandevia, 2012). The density of muscle spindles is distributed diversely across cervical muscles and is particularly high in the small suboccipital muscles, which implies their roles in the fine motor control of the neck (Kulkarni et al., 2001; Boyd-Clark et al., 2002; Liu et al., 2003). The muscle spindles are typically innervated by group Ia and group II afferents, while the GTOs are innervated by group Ib afferents (Jami, 1992; Delhaye et al., 2018). With respect to differences in the anatomical location and type of afferents, the muscle spindles are sensitive to changes in static muscle length and the rate of change in muscle length, while the GTOs are sensitive to the changes in contractile force (Chalmers, 2002; Vincent et al., 2017; Wilkinson, 2022). The core function of the proprioceptors is to transduce mechanical stimulus from muscles and tendons into electrochemical signals and project it *via* dorsal root ganglia (DRG) to the central neural system (CNS) (Delmas et al., 2011; Bewick and Banks, 2015). As a family of mechanosensitive membrane proteins, Piezo channels have been reported to be the main mechanically activated cation channels during this mechanotransduction process (Coste et al., 2012; Murthy et al., 2017). In particular, the expression of the Piezo2 channel is extremely high in DRG sensory neurons (Coste et al., 2010). Additionally, when conditioned with the deletion of Piezo2 channels in proprioceptive neurons, the experimental mice show severe deficits in movement coordination and sensing limb positions (Florez-Paz et al., 2016). Patients with loss of function mutations in the Piezo2 gene display deficits in producing coordinated movements (Chesler et al., 2016; Szczot et al., 2018). However, the exact molecular mechanism of proprioception still needs further research. Proprioceptive sensory afferents typically interact with monosynaptic motor neurons that control the same muscle or synergistic muscles (Manuel and Zytnicki, 2011; Imai and Yoshida, 2018), and neck pain can impaire cervical proprioception and altered motor control strategy of cervical spine (Meisingset et al., 2015, 2016).

### Impaired Proprioception During Neck Pain

Any injuries to cervical structures affect the proprioceptive system, as clearly demonstrated in whiplash-associated and chronic neck patients (De Pauw et al., 2016; Mazaheri et al., 2021). Aside from injuries, cervical structural degeneration that occurs with aging could also lead to proprioceptive deficits (Ferlinc et al., 2019). It has been demonstrated that aged subjects show much fewer intrafusal fibers and denervation of muscle spindles from different parts of the body when compared with young subjects (Swash and Fox, 1972). Studies have confirmed the decline of cervical proprioceptive function in elderly participants (Vuillerme et al., 2008; Landelle et al., 2018), and patients with muscular dystrophy also show spindle morphology changes and corresponding impairment of the proprioception, manifested as postural instability and poor coordination (Kararizou et al., 2007; Troise et al., 2014). Based on literature reviews (Peng et al., 2021), many tests have been applied to measure the sensorimotor control system in neck pain patients, among which the joint position error (JPE) is the most commonly used, reflecting the impairment in joint position sense. Patients with neck pain, in general, show greater JPE when compared with healthy subject, although conflicting results exist between studies due to differences in methodologies (Stanton et al., 2016; de Zoete et al., 2017). In a recent review, the JPE does not differ between patients with traumatic neck pain and non-traumatic neck pain, but both show proprioceptive deficits compared with healthy controls (de Vries et al., 2015). Further, previous studies have also found that cervical JPE was not different between young and old subjects with chronic neck pain (Alahmari et al., 2017). These results indicate that pain itself may have a major influence on the proprioceptive system over degeneration with aging and structural damage to the neck. This point was proved in abundant experimental and clinical neck pain research studies (Malmström et al., 2013; Gizzi et al., 2015; Zaproudina et al., 2015). Furthermore, some previous studies reported that the cervical JPE was positively correlated with neck pain intensity in subjects with cervical spondylosis (Reddy et al., 2019).

Although still unclear, pain may affect cervical proprioception at any stage during the signal transduction process according to the complex neurological pathway (Röijezon et al., 2015). The evidence indicated that the activation of nociceptors (type III and type IV afferents) could inhibit the activity of gamma motor neurons, which leads to proprioceptive disturbance (Riemann and Lephart, 2002; Bennell et al., 2003). Moreover, the cellular bodies of nociceptors are embedded in the dorsal root ganglion as well, and the proprioceptive signals could be competitively suppressed by nociceptive signals in higher CNS centers (Schomburg et al., 1999). Abnormal proprioception from the peripheral cervical structures could cause cortical neuroplastic changes, modify the sensorimotor control system and eventually result in altered motor outputs (Woodhouse et al., 2010; Meisingset et al., 2015; DePauw et al., 2017).

## Sensorimotor Control System

### Sensorimotor Control of the Neck

Three interactive systems are involved in the sensorimotor control of neck movements: the active system (cervical muscles), the passive system (vertebrae, intervertebral disks, ligaments, joint capsules and facet joints) and the central nervous system (Panjabi, 1992a; Izzo et al., 2013). It has been estimated that the mechanical stability of the cervical spine is 20% from the osseoligamentous structures and 80% from the musculature structures (Panjabi et al., 1998). Cervical muscles are the direct performers of the sensorimotor control system, and the coordination between cervical muscles ensures the dynamic stability of the cervical spine during neck movements (Panjabi, 1992b; McGill et al., 2003). More than 20 pairs of cervical muscles surround the cervical spine column, including deep and superficial muscles (Blouin et al., 2007). The deep cervical muscles, typically attached to the cervical vertebrae directly with a small moment during neck movements, are supposed to control individual cervical joint motion (e.g., longus colli, longus capitis, and multifidus muscles) (Blouin et al., 2007; Schomacher and Falla, 2013). By contrast, superficial cervical muscles cross several cervical vertebrae or the entire cervical spine and work as the posture maintainer and movement initiator (e.g., sternocleidomastoid and trapezius muscles) (Blouin et al., 2007; Schomacher and Falla, 2013). Cervical ligaments are traditionally supposed to have only mechanical properties, limiting the cervical joint motion at the extremes of neck movements (Hartman et al., 2016). The ligaments are important passive stabilizers but functionally connected to the surrounding muscles by the ligamento-muscular reflex (Dyhre-Poulsen and Krogsgaard, 2000; Chu et al., 2003; Hendershot et al., 2011). Paraspinal muscles (such as multifidus muscle) could be activated by stimulus in ligaments and restrict the segmental cervical joint motion during neck movements (Solomonow et al., 1998). With respect to a specific movement, the central nervous system continuously collects proprioception feedback and adjusts the motor command to regulate muscle activities and achieve dynamic balance, movement acuity and coordination (Strimpakos, 2011; Röijezon et al., 2015; Qu et al., 2019b).

### Motor Control Strategy During Neck Pain

Neck pain is associated with disturbance in cervical sensorimotor control (Woodhouse and Vasseljen, 2008; Gizzi et al., 2015). The motor control strategy of the cervical spine has been most commonly studied by measuring electromyographic (EMG) activity of the cervical muscles involved in a specific motor task (Falla and Farina, 2008). The structural complexity of the cervical spine reflects its potential compensatory mechanism under pathologic conditions (Vasavada et al., 2002; Falla and Farina, 2008). In experimental neck pain studies, the same submaximal-load motor task could be accomplished in the presence of pain by reorganizing the activation of the cervical muscles involved (Tucker et al., 2009; Muceli et al., 2014; Abboud et al., 2016). This kind of reorganization strategy exists between different parts of the same muscle or muscle groups involved in the task (Falla et al., 2007b; Falla and Farina, 2008; Samani et al., 2009). In principle, the CNS explores control strategies to complete the same motor task by minimizing the use of the painful muscle in order to reduce further pain or injuries (Falla, 2004; Falla et al., 2007a). Therefore, the painful muscle generally shows decreased EMG activity during the motor task, together with redistribution of activation among the synergist and antagonist muscles (Falla and Farina, 2007, 2008; Falla et al., 2007a). The altered motor control strategy, in consequence, is often task-specific and direction-specific due to the role of the painful muscles (agonist or antagonist) in the task (Falla et al., 2007a).

Patients with neck pain are typically associated with decreased activity of deep cervical muscles and increased activity of superficial cervical muscles (Schomacher and Falla, 2013; Tsang et al., 2014). In addition, enhanced cervical muscle co-activation has also been demonstrated in previous studies, which is considered to be a strategy to increase the stiffness of the cervical spine (Cheng et al., 2014). This finding aligns with previous studies showing that the cervical spine is controlled in a more stiffening pattern with neck pain (Meisingset et al., 2015). Delayed onset of activation, prolonged activation and reduced resting periods are the other manifestations of deep cervical muscles in patients with neck pain (Falla et al., 2004a,b).

### Quantitative and Qualitative Kinematics With Neck Pain

The deficit in the sensorimotor control system alters the kinematic characteristics of the cervical spine in patients with neck pain, including both the quantitative and qualitative aspects, which have been widely reported in previous studies (Ylinen et al., 2004; Sjölander et al., 2008; Sarig Bahat et al., 2010; Tsang et al., 2013). The quantitative measurements reflect the ability of the neck to achieve a specific motor task, such as maximal voluntary contraction (MVC) and cervical range of motion (ROM), which are reported to be reduced in patients with neck pain if beyond the compensatory capacity of the cervical spine (Lindstroem et al., 2012; Rudolfsson et al., 2012). On the other hand, the qualitative parameters indicate the quality of the motor task execution and more representatively reflect the altered motor control strategy during the motion process with neck pain. The velocity, acceleration, smoothness, accuracy, conjunct motion, and ROM-variability of neck movements have been demonstrated to be different between patients with neck pain and healthy controls (Sjölander et al., 2008; Sarig Bahat et al., 2010). However, the quantitative and qualitative measurements both showed conflicting results in previous studies or reviews, which may result from methodologic differences and sample bias et al. (Kauther et al., 2012; Franov et al., 2022). The above-mentioned parameters are gross motor outputs and cannot reflect the individual cervical joint impairment. Meanwhile, the motor deficit of an individual joint will be compensated by the other joints due to the compensative mechanism within the cervical spine resulting in unchanged motor outputs (Schwab et al., 2006; Lan et al., 2014). Theoretically, the altered motor control strategy during pain could change tissue loading, the direction and magnitude of joint forces and contributes to the altered cervical joint motion patterns (Yoganandan et al., 2001). The motor impairments are sometimes subtle and cannot be detected by traditional physical examination (Oddsdottir and Kristjansson, 2012). New dynamic motion parameters, such as anti-directional joint motion or joint motion variability, are needed to precisely capture this motor alteration at individual cervical joints (Qu et al., 2019a,b, 2020).

## Neural Plasticity

### Neural Plasticity and Proprioception

The ability of neurons to change function, form and number is called neural plasticity (Citri and Malenka, 2008). Adaptive neural plasticity results in changes in the synaptic connection strength between neurons under physiological conditions, and it is a critical process for improving brain functioning (Citri and Malenka, 2008). It is, for example, an essential neuronal substrate for learning and memory (Pascual-Leone et al., 1994). Maladaptive neural plasticity is the pathological side of adaptative neural plasticity and is caused by an imbalance in the synaptic activity of the nervous system (Kuner and Flor, 2017). The effect of maladaptive neural plasticity is a loss of nervous system coordination and function, resulting in impairment and deterioration in the quality of life. Maladaptive neural plasticity during prolonged and persistent pain has been suggested in recent years, and it has been proposed that sustained nociceptive inputs from an injured tissue might result in dysfunctional neural plasticity changes (Kuner and Flor, 2017). Based on various neurophysiological and neuroimaging studies, dysfunctional nervous system activity (Tsao et al., 2011), coupled with structural remodeling (Mansour et al., 2013; Baliki and Apkarian, 2015), has been reported in individuals suffering from persistent musculoskeletal pain, including neck pain (DePauw et al., 2017).

### Maladaptive Neural Plasticity and Neck Pain

Clinically, somatosensory, proprioceptive and neuromuscular impairments are commonly reported in patients with chronic neck pain. Some of these impairments include cold and mechanical pain hyperalgesia in the neck region (Johnston et al., 2009; Walton et al., 2011), forward head posture (Mahmoud et al., 2019), altered joint motion pattern (Qu et al., 2020), and dysfunction of the deep cervical flexor muscles (Falla et al., 2004b). Patients with chronic neck pain also tend to show unsuitable emotional and cognitive factors associated with pain, such as pain catastrophizing and fear of movement (Dimitriadis et al., 2015; Lee et al., 2015), and nociceptive pain episodes increase the probability of becoming chronic pain when various psychosocial variables exacerbate maladaptive processes triggered by pathophysiological factors (Kuner and Flor, 2017). Since, in many patients with neck pain, particularly those with chronic symptoms, a clear pathophysiological origin explaining the experience of pain is lacking (Elliott et al., 2009), or the nociceptive source is not significant enough to justify the neck pain reported by patients, researchers have moved the focus away from abnormal musculoskeletal tissue explanations and started exploring the role of the nervous system, such as central sensitization (Latremoliere and Woolf, 2009; Peirs and Seal, 2016). Central sensitization mainly occurs due to persistent peripheral nociceptive stimulation, is reported to contribute to the chronic pain and mainly depends on neuronal changes in the CNS (Ji et al., 2018; Bonanni et al., 2022). In some of those patients, there is frequently clinical evidence of maladaptive pain neural plasticity (Van Oosterwijck et al., 2013), a general term used to indicate an alteration in the function of neurons and circuits in nociceptive pathways (Lefaucheur et al., 2014). In the last few decades, the involvement of the nervous system in chronic pain conditions has been widely explored using electrophysiological and imaging techniques (Kuner and Flor, 2017). For instance, from a sensory perspective, reorganization of the primary somatosensory cortex has been examined in patients affected by chronic low back pain using magnetoencephalography (Flor et al., 1997). Motor-evoked potentials (MEPs) to transcranial magnetic stimulation (TMS) have also demonstrated a smudging of corticospinal excitability of specific muscles (overlap of motor cortical maps and centers of gravity) in individuals affected by persistence/recurrence of low back pain compared to healthy control (Tsao et al., 2008; Schabrun et al., 2017). These results may indicate that the primary somatosensory cortex and motor corticospinal excitability show maladaptive neural plasticity in people affected by musculoskeletal pain, including chronic neck pain. Furthermore, neuroimaging studies have also demonstrated that emotional and cognitive regions of the brain, such as the medial prefrontal cortex, amygdala and hippocampus (Mutso et al., 2014; Baliki and Apkarian, 2015), are altered in chronic musculoskeletal pain patients, suggesting that these regions may also be critically involved in the abatement of chronic neck pain.

## Interventions

### Sensorimotor Control System Training

Exploring the effective treatment of neck pain has long been a challenge. For the importance of the sensorimotor control system, treatments aiming to restore sensorimotor function have been proposed as important managements of neck pain, including balance exercise, joint position and movement sense training, gaze direction recognition exercise, sensory discrimination training, and coordinative exercises (Beinert and Taube, 2013; Kälin et al., 2016; Duray et al., 2018; Saadat et al., 2019). These treatments, in essence, either enhance position/motion sense by repeatedly provoking the proprioceptors or correct motor patterns by increasing the targeted muscle activity (Peng et al., 2021). Abundant evidence has revealed that the proprioceptive training and motor control exercises could improve the joint reposition accuracy and neck disability, and reduce the pain intensity in patients with neck pain, although treatment methods vary among studies (Beinert and Taube, 2013; Sarig Bahat et al., 2015; Duray et al., 2018; Saadat et al., 2019). In a balance exercise, subjects typically need to keep their head upright when standing by a single leg or on a wobble board with/without visual feedback. Beinert and Taube et al. found that the balance exercise can reduce pain intensity and improve the JPE in patients with neck pain (Beinert and Taube, 2013). Gaze direction recognition exercise is able to enhance the beneficial effect of conventional physical therapy on pain reduction, functionality recovery and balance performance (Duray et al., 2018). Deep cervical flexor and extensor training are reported to reduce pain intensity and functional disability in patients with chronic mechanical neck pain, but the effect on strength and endurance remain conflicting (Blomgren et al., 2018; Suvarnnato et al., 2019). Coordination exercises, aiming to restore the active neck movements and retrain the fine movement control of the cervical spine, are reported to reduce pain and alter motor control strategy between deep and superficial cervical muscles (Rudolfsson et al., 2014). With the development of virtual reality (VR) techniques, the VR-based kinematic training on patients with neck pain shows improvements in range of motion, accuracy, velocity, smoothness, fine motor control and coordination of the cervical spine (Nusser et al., 2021). It is believed that the VR-based kinematic training could motivate the visual systems, vestibular systems and sensorimotor control system simultaneously in patients with neck pain (Sarig Bahat et al., 2015). Furthermore, the VR-based training method shows an effect on overcoming kinesiophobia in patients with neck pain (Tejera et al., 2020). However, no conclusion could be made that the sensorimotor therapy is better than other kinds of treatments since there is no unification in terms of interventions, therapy time, populations and variety of control groups across research studies (McCaskey et al., 2014). The beneficial effects of proprioceptive training could be augmented when combined with other therapy exercises, such as physical exercises and biofeedback (Sielski et al., 2017; Saadat et al., 2019; Tsiringakis et al., 2020). Therefore, more large samples of randomized controlled trials are needed to provide robust evidence on sensorimotor control system training. Evidence has shown that proprioceptive training is associated with reorganization within the sensorimotor cortex (Aman et al., 2014). Previous studies indicate that the sensorimotor therapies may reverse the pain-induced cortical changes to a normal level based on the plasticity property of the nervous system, which partially explains the symptoms relief and functions recovery in patients with neck pain (Moseley and Flor, 2012).

### Modulation of Maladaptive Neural Plasticity in Neck Pain

Based on electrophysiological and neuroimaging findings in chronic pain patients, treatments that reverse maladaptive neural plasticity, such as non-invasive brain stimulation techniques, have been proposed as a substantial potential for improving future rehabilitation processes (Schabrun and Chipchase, 2012). Non-invasive brain stimulation techniques utilize electromagnetic principles to modulate neural activity non-invasively by generating cortical electrical fields (Rossini et al., 2015). Two main classes of non-invasive brain stimulation are currently applied for research and clinical purposes: repetitive transcranial brain stimulation (rTMS) and transcranial electrical stimulation (tES). Both techniques have generally been shown to be partially effective in reducing pain for some non-musculoskeletal pain conditions, such as peripheral neuropathic pain and migraine, and musculoskeletal pain conditions, such as low back pain (Lefaucheur et al., 2014). However, the clinical evidence for non-invasive brain stimulation in chronic neck pain is still lacking, although some preliminary modulatory effects on motor cortex excitability and analgesic effects have been proven in chronic low back pain (Schabrun et al., 2014; Ambriz-Tututi et al., 2016).

A recent meta-analysis demonstrated that non-invasive brain stimulation increased pain thresholds across all modalities, including mechanical and thermal, in healthy individuals when pooling studies of rTMS and tES of the primary motor cortex (Giannoni-Luza et al., 2020). A recent randomized controlled trial in individuals with chronic low back pain looked at the efficacy of rTMS (Ambriz-Tututi et al., 2016), and by the third week of treatment, 41 patients who received 20-Hz rTMS stimulation over the primary motor cortex showed an 80% reduction in pain from baseline, which was considerably lower than those who received sham rTMS. Pressure pain thresholds also increased in healthy individuals following daily sessions of rTMS on the left dorsolateral prefrontal cortex (De Martino et al., 2019a). In a sham-controlled design study, daily rTMS sessions targeting the left dorsolateral prefrontal cortex reduced long-term pain intensity induced by intramuscular nerve growth factor injections, as well as reversing pain-induced pressure hyperalgesia, altered cortical somatosensory excitability, and corticomotor excitability (Seminowicz et al., 2018; De Martino et al., 2019b). Similar analgesic findings were observed following rTMS to the primary motor cortex in a similar long-term pain paradigm (Cavaleri et al., 2019). This proof of concept demonstrates the use of rTMS in larger musculoskeletal pain studies, and, with more research and a stronger focus on clinical outcomes, it is possible that rTMS may become an integral part of the treatment arsenal for therapists for chronic neck pain in the future.

Using non-invasive brain stimulation to the primary motor cortex has also been shown to augment motor training-induced plasticity by producing a rapid and powerful after-effect in facilitating or depressing the motor cortex excitability, outlasting the stimulation period (Bolognini et al., 2009). Although the mechanics are still unclear, non-invasive brain stimulation techniques may cause different patterns of calcium influx to postsynaptic neurons through N-methyl-D-aspartate channels and gamma-aminobutyric acid receptors, resulting in long-term potentiation and long-term depression in the motor cortex (Huang et al., 2017). There is preliminary evidence that therapies aimed at motor control can improve motor cortex excitability and alleviate pain in chronic musculoskeletal disorders. For example, in the case of chronic low back pain, interventions targeting the primary motor cortex representation of lumbar multifidus muscles or the primary somatosensory cortex of the low back could alleviate pain symptoms (Flor et al., 2001; Moseley et al., 2008; Tsao et al., 2010, 2011). Because sensorimotor skill training changes the motor cortex excitability (Pascual-Leone et al., 1995, 2005), therapeutic techniques targeting the primary motor cortex may be able to restore optimal muscle function. However, to date, no studies have investigated the effect of rTMS on the cervical motor output and it is still unknown whether rTMS can produce changes in the cervical motor control strategy.

## Summary and Outlooks

Altered cervical sensorimotor control system and maladaptive neural plasticity are likely to play a major role in chronic neck pain, and, consequently, various clinical assessments and treatments have been proposed. However, previous research has found conflicting results when these assessments or treatments have been applied to patients with neck pain, likely due to no established standardization. The molecular mechanism underlying proprioception needs to be clarified in the future, which may help to develop mechanism-based therapies for neck pain. New precise motor parameters reflecting sensorimotor deficit and more effective interventions targeting sensorimotor control system or neural plasticity are encouraged to be proposed.

## Author Contributions

All authors listed have made a substantial, direct, and intellectual contribution to the work, and approved it for publication.

## Conflict of Interest

The authors declare that the research was conducted in the absence of any commercial or financial relationships that could be construed as a potential conflict of interest.

## Publisher’s Note

All claims expressed in this article are solely those of the authors and do not necessarily represent those of their affiliated organizations, or those of the publisher, the editors and the reviewers. Any product that may be evaluated in this article, or claim that may be made by its manufacturer, is not guaranteed or endorsed by the publisher.
